# Applying negative rule mining to improve genome annotation

**DOI:** 10.1186/1471-2105-8-261

**Published:** 2007-07-21

**Authors:** Irena I Artamonova, Goar Frishman, Dmitrij Frishman

**Affiliations:** 1 Institute for Bioinformatics, GSF – National Research Center for Environment and Health, Ingolstädter Landstraße 1, 85764 Neuherberg, Germany; 2 Group of Bioinformatics, Vavilov Institute of General Genetics RAS, Gubkina 3, 119991 Moscow, Russia; 3 Department of Genome Oriented Bioinformatics, Technische Universität Munchen, Wissenschaftzentrum Weihenstephan, 85350 Freising, Germany

## Abstract

**Background:**

Unsupervised annotation of proteins by software pipelines suffers from very high error rates. Spurious functional assignments are usually caused by unwarranted homology-based transfer of information from existing database entries to the new target sequences. We have previously demonstrated that data mining in large sequence annotation databanks can help identify annotation items that are strongly associated with each other, and that exceptions from strong positive association rules often point to potential annotation errors. Here we investigate the applicability of negative association rule mining to revealing erroneously assigned annotation items.

**Results:**

Almost all exceptions from strong negative association rules are connected to at least one wrong attribute in the feature combination making up the rule. The fraction of annotation features flagged by this approach as suspicious is strongly enriched in errors and constitutes about 0.6% of the whole body of the similarity-transferred annotation in the PEDANT genome database. Positive rule mining does not identify two thirds of these errors. The approach based on exceptions from negative rules is much more specific than positive rule mining, but its coverage is significantly lower.

**Conclusion:**

Mining of both negative and positive association rules is a potent tool for finding significant trends in protein annotation and flagging doubtful features for further inspection.

## Background

There are currently over six million amino acid sequences known, and only a quarter of a million have been manually annotated [1]. Moreover, as estimated by [2], merely for 3% of proteins functional annotation is based on experimental evidence. Due to the advent of super-fast and ultra-low-cost DNA sequencing technologies [3] the speed of genome sequencing continues to increase, and sequencing prices continue to plummet. Given the quickly widening gap between the amount of molecular data and the capacity of human experts there is little doubt that electronic annotation by automated software pipelines will be the only source of information about the overwhelming majority of proteins.

*In silico *annotation generated by bioinformatics methods has the advantage of being efficient and cheap, but at the same time suffers from a notoriously high error level [4,5]. Most of these errors are caused by homology-based annotation transfer where available similarity is not sufficient to warrant the transfer of information from the source to the target sequence, or because the annotation of the source sequence is already wrong. Further complications include the presence of compositionally biased sequence regions, mosaic domain structure of eukaryotic proteins, wrong gene models, and difficulties in recognizing pseudogenes.

The most obvious and direct approach towards improving the reliability and coverage of unsupervised protein annotation entails the development of better bioinformatics tools. Remarkable algorithmic advances of the past decade include more accurate gene prediction techniques (reviewed by [6]), highly sensitive sequence similarity searches based on hidden Markov models [7], protein secondary structure prediction approaching the 80% accuracy barrier (e.g., [8]), novel tools for predicting protein cellular localization [9], improved strategies for annotation transfer by homology (e.g., [10]), and enhanced protein function prediction by phylogenomics methods [2], to name just a few. Automatic annotation efforts also significantly profit from highly curated and actively maintained information resources, such as sequence [1], pathway [11] and interaction [12] databases, functional and structural classifications of proteins [13-15], as well as the collections of protein domains and motifs [16,17].

A complementary tactic to improve the quality of protein sequence databases involves retrospective search for errors in the total corpus of already available annotation. Under this approach protein annotation is considered to be a collection of records, one per each gene, containing a varying number of attributes, ranging from just a few minimal descriptors (length, pI) for hypothetical proteins, to dozens of annotation items (motifs, EC numbers, localization, structural folds, etc) for better characterized proteins. Modern data mining techniques can be used to identify statistically significant associations between individual attributes, and then to investigate exceptions from such associations that can potentially point to erroneous assignments.

In our earlier work we applied the formalism of association rule mining to extract associations between annotation items in large molecular sequence databases [18]. Considering a database with multiple entries, with each entry ascribed a finite number of features, association rules [19] are simple implications that can be formulated in the form (A_1 _& ... & A_n_) => Z, where A_1 _... A_n _(the left-hand side of the rule, LHS) and Z (right-hand side, RHS) are different features, and the rule means "database entries that possess all features A_1 _... A_n _are likely to possess feature Z". The rules of this type are thus *positive *because they model a positive relation between two item sets. Each rule is characterized by its coverage, the number of entries in the database that possess all features A_1 _... A_n_, its support, the number of entries satisfying both the left and the right sides of the rule simultaneously, and its strength, which is essentially the probability that a given database entry will satisfy the right side of the rule given that it satisfies the left side of the rule.

Our strategy for finding errors in annotation consisted of finding rules with a strength very close, but not equal, to 1.0, which means that such rules have a minor number of exceptions, and then identifying all proteins that constitute exceptions to these rules. Applied to the Swiss-Prot [1] database, this approach yielded 7396, 4956, and 4046 rules with a strength greater than 0.95 and a coverage of over 50 which were not fulfilled exactly once, twice, or three times. In order to test whether exceptions from strong rules actually correspond to annotation errors subsequent releases of the Swiss-Prot database were compared and additional manual verification was conducted. It was indeed found that exceptions to strong rules get corrected substantially more often than the rest of the annotation. For unsupervised annotation automatically generated by the PEDANT genome analysis system [20] the total fraction of exceptions from strong rules classified by manual analysis as errors was as high as 68%. It was also found that most of the errors in the Swiss-Prot database are under-predictions (i.e. absence of features that would be expected based on association rules), consistent with the prudent manual annotation process adopted by Swiss-Prot, while in PEDANT errors are typically caused by over-predictions.

In this work we continue to explore the application of rule mining to correcting annotation errors and investigate the utility of negative association rule mining, which, as the name implies, represents the identification of negative relationships between item sets [19]. A negative association rule has the form A_1 _& ... & A_n _(LHS) => not Z (RHS), with A_1 _... A_n _and Z being different features, and the rule means '*database entries that possess all features *A_1 _... A_n _*are unlikely to possess feature *Z'. For negative rules *support *is the number of database entries satisfying both the LHS and the RHS, i.e. those entries that possess all features A_1 _... A_n _and do not possess the feature Z. An additional very important parameter used in this work to characterize negative rules is *leverage *which is defined as the difference of the rule support and the product of supports of its LHS and RHS. Leverage measures the unexpectedness of a rule as the difference of the actual rule frequency and the probability of finding it by chance with the given frequencies of its RHS and LHS.

A negative association rule is thus an implication from the union of several items to an item negation. An example of a trivial biologically relevant negative association rule is "Nuclear localization => not bacterial origin", i.e. every protein annotated as localized in the nucleus cannot have a bacterial origin. As with positive rules, negative rules are not necessarily absolutely strict. For instance, the rule "Operon structure => not eukaryotic origin" has a number of exceptions because bacterial-like operons were described in *Ceanorhabditis elegan*s [21]. Since these exceptions comprise only a small fraction of the annotated genes, this rule may naturally be interpreted as "the majority of genes constituting an operon structure do not originate from eukaryotic organisms". In this specific case exceptions from a strong rule are biologically motivated and do not represent errors. However, in many other cases such exceptions do point to erroneously assigned annotation items. For example, the rule "intracellular transport vesicles => not bacteria" has four exceptions in the PEDANT annotation caused by erroneous homology-based transfer of the functional category "intracellular transport vesicles" to four bacterial gene products. We present a large-scale evaluation of exceptions from strong negative rules in the PEDANT genome database and assess their utility for detecting and correcting annotation errors. To our knowledge this is the first application of negative association rule mining to molecular biological data.

## Methods

### Extracting item sets from the PEDANT genome database

PEDANT software suite [20] is an automatic annotation pipeline that runs various bioinformatics analyses on each protein sequence and stores the results, appropriately parsed, in a relational database. The associated PEDANT genome database [22] currently contains pre-computed annotation for 468 genomes and a total of more than 1.76 million gene products. Most of these data were calculated using the version 2 of the PEDANT software, but we are currently deploying the all-new version 3 with substantially enhanced capabilities including a new graphical user interface and an extended set of bioinformatics algorithms.

Here we used the following 10 model genomes freshly processed using PEDANT3 (see ): *Helicobacter pylori*, *Arabidopsis thaliana*, *Saccharomyces cerevisiae*, *Thermoplasma acidophilum*, *Synechocystis sp*., *Parachlamydia*, *Mycobacterium tuberculosis*, *Aeropyrum pernix*, *Escherichia coli*, and *Bacillus subtilis*. The total of 55063 gene products were annotated with more than 1 million (1265974) annotation features suitable for association rule mining (see Table 1).

**Table 1:** Annotation features used in this work

			Method used						
								
Feature name	Description	Examples	Algorithm	Threshold value used	Reference	Number of proteins having items of this type	Total number of items found	Average number of items per protein	Total number of attribute values
Length	Protein length (number of amino acids) binned over four ranges	Small (<120), Medium (>=120, <1000), Large (>=1000, <1500), eXtraLarge (>=1500)	Direct calculation	Not applicable	None	All (55063)	All (55063)	1	4
GC content of the gene	The value of the GC-content binned over 3 ranges	L (<=0.4), M (<0.5), H (>=0.5)	Direct calculation	Not applicable	None	30218*	30218	1	3
Isoelectric point	The value of the isoelectric point binned over 4 ranges	C (aCid, pI <=5.5), NC (Neutral-aCid, 5.0 < pI <=7.0), NL (Neutral-aLkaline, 7.0 < pI <=9.2), L (aLkaline, pI > 9.2)	Direct calculation	Not applicable	None	All (55063)	All (55063)	1	4
Low complexity regions	Percentage of residues predicted to be in low complexity regions binned over three ranges	High (>=10%), Medium (0–10%), None (0%)	SEG	Default SEG parameters	(Wootton, 1994)	All (55063)	All (55063)	1	3
Disordered regions	Percentage of residues in disordered regions binned over 4 ranges	High (>=20%), Medium (10–20%), Low (0–10%), 0 (0%)	DisEMBL	Default DisEMBL parameters	(Linding et al., 2003)	All (55063)	All (55063)	1	4
Coiled coil regions	Presence of coiled coil regions	COILS:+	COILS	Default COILS parameters	(Lupas, 1997)	7809	7809	1	1
Structural class derived from secondary structure prediction	Classification of proteins based on the prevalent type of secondary structure	Alpha/beta	Predator	Default Predator parameters	(Frishman and Argos, 1997)	52711	52711	1	4
Transmembrane segments	Presence and number of transmembrane segments	TM (=transmembrane domains are present), 1 TMs, 12 TMs (the number of TM domains)	TMHMM	Default TMHMM parameters	(Krogh et al., 2001)	12437	24874	2	52
Signal peptide	The presence of the signal peptide	SignalP:+	SignalP	Default SignalP parameters	(Bendtsen et al., 2004)	8066	8066	1	1
Protein localiza-tion	Predicted cellular localization	Secretory pathway	TargetP	Default TargetP parameters	(Emanuelsson et al., 2000)	18186	18186	1	2
*SCOP super-families*	*Classification of proteins into superfemilies based on their tertiary structure, corresponds to the third level of the SCOP hierarchy*	*a.47.3 (Cag-Z)*	*RPS-BLAST*	*E-Value 1E-10*	*(Lo et al., 2002)*	*29562*	*37360*	*1.26*	*1096*
*Interpro*	*Sequence domains found by HMM profile searches: a. primary domains; b. IPR domains*	*IPR003593 (AAA_ATPase domain), PF02985 (PFAM primary domain, HEAT repeat)*	*BLASTP*	*E-Value 1E-10 InterPro-Scan*	*Putin et al. (2006)*	*a. 43829 b. 42627*	*a. 142433 b. 83227*	*a. 3.25 b. 1.95*	*18106*
*EC numbers*	*Enzyme Commission Classification of enzymatic activities*	*Ec1.1.1.1*	*BLASTP*	*E-Value 1E-10*	*(Webb, 1992)*	*11869*	*15610*	*1.32*	*1753*
*COG*	*Ortologous groups of genes from for prokaryotic and eukaryotic organisms organisms)*	*COG0582 (Integrase), KOG1327 (Copine)*	*RPS-BLAST*	*E-Value 1E-10*	*(Tatusov et al., 2003; Koonin et al., 2004)*	*33930*	*50272*	*1.48*	*8048*
*Keywords*	*Swiss-Prot or PIR keywords*	*Ligase*	*BLASTP*	*E-Value 1E-10 BLASTP*	*(Wu et al., 2006), (Wu et al. 2002)*	*20128*	*80910*	*4.02*	*632*
*Functional categories*	*Two upper levels of the MIPS Functional Catalog*	*Fc40.20 (Cell fate: aging)*	*BLASTP*	*E-Value 1E-10*	*(Ruepp et al., 2004)*	*32248*	*438699*	*13.60*	*171*

* For technical reasons GC content values were not available for *Arabidopsis thaliana *genes at the time of writing.

Features transferred by similarity (type 3, see Methods) are shown in italic.

A typical annotation entry extracted from PEDANT for a given gene product has the following form:

"length:S, Pi:C, GC:H, Bacteria, alpha/beta, do:L, *b.129.1*, *fc16.03*, lc:0, *fc16*, *fc40.01*, *PF04014*, COG2336, *IPR007159*, *fc40, DNA-binding, fc32, fc32.05*"

This line describes the antitoxin of the ChpB-ChpS toxin-antitoxin system from *Escherichia coli *as a protein that has small length (length:S), acidic isoelectric point (Pi:C, less than 5.5), gene with high GC-content, bacterial origin (Bacteria), and low content of disordered regions (do:L), does not possess any low complexity regions (lc:0), has structural class of the 'alpha/beta' type and the PFAM [16] domain *PF04014*. It belongs to the *IPR007159 *InterPro [23] family and the *b.129.1 *SCOP [13] structural superfamily, and it is a homolog of the UniProt [1] proteins annotated with the keyword *"DNA-binding"*. According to the MIPS Functional Catalog [15] (only two upper levels are considered here) the function of this protein is described by the labels *fc16 *(protein with binding function), *fc16.03 *(nucleic acid binding), *fc40 *(cell fate), *fc40.01 *(cell growth/morphogenesis), *fc32 *(cell rescue, defense and virulence) and *fc32.05 *(disease, virulence and defense).

Annotation attributes extracted from PEDANT can generally be subdivided into three types in terms of their intrinsic susceptibility to errors.

• Type 1. Features that are definitely known. This group includes either inherent properties of genes and their products, such as their taxonomic origin, or features that can be unambiguously calculated from primary sequences, such as GC content, length, pI value, percentage of low complexity regions, and so on.

• Type 2. Structural and functional properties of proteins predicted directly from their amino acid sequences by *ab initio *computational algorithms (secondary structure, disordered regions, coiled coils, transmembrane segments, signal peptides, cellular localization).

• Type 3. Structural and functional properties of proteins derived by similarity searches against previously characterized gene products. These features include sequence domains, keywords, functional categories, enzyme classes, and functional and structural superfamilies.

It is obvious that the features of type 1 are unfaultable and cannot generally contain errors (except for incorrectly predicted gene models, typographical errors, or errors caused by software bugs or human error). Features of type 2 are typically predicted with the accuracy in the order of 70% [24] by machine learning techniques, such as neural networks or support vector machines. If no experimental data for a given feature type is available (e.g. known three dimensional structure, experimentally determined cellular localization), such predictions can only rarely be further improved by human curation. Finally, features of type 3 are transferred from one or several previously annotated gene products to the query protein based on a sufficiently significant degree of similarity. These features constitute the main bulk of protein-associated information available in the databases, and it is precisely this part of protein annotation that is especially prone to errors due to intrinsic limitations of annotation transfer by homology.

We are interested in applying negative association rule mining for identifying errors in the annotation attributes of Type 3 transferred by similarity from other proteins; in the annotation entry above such features are shown in italic. In our dataset there were the total of 848511 similarity-derived features (67% of all features analyzed), more than a half of which were constituted by functional category assignments.

### Extracting rules from PEDANT annotation

The annotation set describing 55063 genes in ten PEDANT genomes served as input data to extract negative association rules using a modified version of the well-established *Apriori *algorithm for association rule mining. The basic *Apriori *algorithm, described in detail in [25], is designed to find frequent item sets by consecutive expansion of candidate sets at every step. It is based on the simple notion that all subsets of a frequent item set are also frequent. In this work we used a version of this algorithm designed for the efficient negative association rule production [26] implemented and kindly provided by Christian Borgelt (for initial software description see, e.g., [27]).

The application of the *Apriori *software to PEDANT annotation results in a file containing one negative rule per line. Each line lists the LHS and the RHS as well as several numerical characteristics of the rule delimited by commas. A typical rule line in the output file looks like this:

"fc34.11 & fc36, not length:S, 0.028, 1560, 0.895, 49286, 0.028, 1558, 0.999, 1.116, 0.003, 161.669"

This notation means that proteins possessing FunCat labels 34.11 ("Cellular sensing and response") and 36 ("Interaction with the environment (systemic)") are unlikely to be of small (less than 120 amino acids) length. The LHS items are joined by the "&" symbol and are followed by the RHS (here – a negation of the annotation feature), and the list of numerical attributes of the rule: coverage, coverage count, RHS coverage, RHS coverage count, support, support count, strength, lift, leverage, and leverage count. In addition to "Support count", "Coverage count" and "Strength", important for positive association rule selection [18] (1560, 1558 and 0.999, respectively, in our example), the numerical parameter "Leverage" (or "interest of the rule", as alternative name) is also very important in the case of negative rules. All existing algorithms allow calculating negative association rules effectively only using a certain threshold on the minimal leverage or leverage count. Here, if not specified otherwise, all rules with the support and the leverage counts of at least 100 proteins and strength of at least 0.1 were retained for further analysis.

### Analysis of taxon specificity

A considerable and arguably the most valuable part of PEDANT annotation involves assignments of functional categories based on the MIPS FunCat [15]. A large fraction of negative association rules included a taxon-specific FunCat label (e.g., fc75.03 – "animal tissue") on one side of the implication and the taxon of protein origin contradicting this specificity (in the given case, Bacteria or Archaea or Viruses for fc75.03) on the other. Among all 184 different FunCat labels (2 upper levels of the hierarchy) used in this study 71 were taxon-specific. For example, one of the taxon-specific rules found was:

"fc75.03 & fc34.11 & fc10.03 & fc20, not Bacteria, 0.006, 344, 0.647, 35620, 0.006, 342, 0.994, 1.537, 0.002, 119.467".

All rules of this kind are a direct consequence of the taxon-specific nature of the underlying (here fc75.03) FunCat labels. Some of these rules may have exceptions due to annotation transfer by homology between proteins from different taxonomic groups. We classify such cases as annotation errors according to the general procedure.

### Manual verification of the rules

For manual verification of negative association rules we randomly selected a limited sample of protein entries from the PEDANT annotation set that constituted exceptions from rules and could not be corrected by the taxon specific analysis explained in the previous section. Annotation features of these proteins occurring either in the LHS or in the RHS of the rules were subjected to careful manual analysis by an experienced protein annotator according to the established procedures routinely used at MIPS for genome annotation (see, e.g., [28]). These include assessment of similarity hits and predicted protein features as well as in-depth examination of biological literature describing experimental studies. An exception was classified as an error if one of the features in the LHS or RHS of the rule was found to be assigned wrongly to the given protein entry. We then calculated the error rate among all manually analyzed exceptions according to the following formula:

(percentage of exceptions classified as annotation errors among all manually verified exceptions * number of exceptions in rules not involving 'taxon specificity' + number of exceptions from 'taxon specific' rules)/overall number of exceptions.

### Manual verification of annotation features

We filtered out wrongly assigned taxon-specific FunCat labels and selected randomly a limited sample among all remaining homology-transferred annotation features. The accuracy of the feature assignment was thoroughly verified by an experienced annotator. All verified annotation attributes were divided into 3 categories: true assignments, false assignments, or "not known". The latter category was selected if the evidence for a given assignment was not sufficient to make a judgment, but the feature did not obviously contradict the nature of the protein (e.g., the keyword "Zymogen" in the annotation of the lysosomal Pro-X carboxypeptidase from Arabidopsis thaliana, code "At5g65760"). Features of this category were excluded from further analysis and were not taken into account while estimating the error level. For example, if in a set of 100 features selected for manual verification 40 features were classified as 'errors', 56 as 'correct assignments', and 4 as 'not known', then the final estimate of the error level in this sample was 100*40/(100-4) = 42%.

## Results and discussion

### Statistics of negative association rules in the PEDANT annotation

Application of the *Apriori *algorithm to the annotation set extracted from PEDANT resulted in 9591 negative rules (see Additional file 1). For example, one of the most trivial rules found was "Bacteria, not Eukaryota, 0.353, 19443, 0.413, 22765, 0.353, 19443, 1.000, 2.419, 0.207, 11404.573". This rule is satisfied in all possible cases and thus its strength is 1.0 with no exceptions. In total there were 2273 such rules calculated. Much more interesting rules in the context of this study are those of strength very close, but not equal to 1.0. These rules have a small number of exceptions that may constitute annotation errors. There were 7318 such rules with 26969 exceptions in total. An example of such rules is "Nuclear protein, not Bacteria, 0.033, 1808, 0.647, 35620, 0.033, 1798, 0.994, 1.537, 0.011, 628.413". This statement which is obvious from the biological point of view nevertheless does not make an absolute rule; in fact out of all 1808 protein entries annotated by the keyword "Nuclear protein" in the PEDANT database only 1798 actually have eukaryotic origin. The ten proteins constituting exceptions from this rule (for example, the oligoribonuclease from *Mycobacterium tuberculesis*, PEDANT code gi_15609648) simply inherit this keyword from their eukaryotic homologs. In our example, the homolog is human oligoribonuclease (PEDANT code gi_116242694, UniProt code ORN_HUMAN), one of the alternatively spliced isoforms of which is localized to the nucleus.

**Figure 1:**
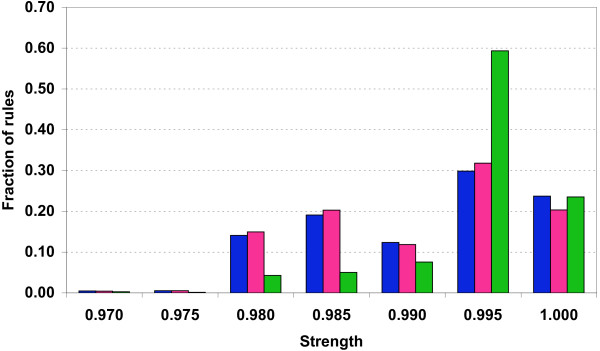
**Distribution of negative association rule strength **(probability that a given database entry will satisfy the right side of the rule given that it satisfies the left side of the rule). Minimal coverage counts (number of entries in the database that possess all features from the left hand side of the rule) used are 100 (blue), 200 (pink), and 500 (green). The threshold for minimal leverage count (difference of the actual rule frequency and the probability to find it by chance with the given frequencies of its RHS and LHS) was set to 100 in all calculations

Some aspects of negative rule statistics differ significantly from positive association rules due to vastly different item frequencies. Because annotation items themselves are rare, and most items are in fact extremely rare (e.g., the PFAM domain PF01029 is only found in 12 (0.02%) of proteins analyzed), their negations used in negative association rule mining are unavoidably very frequent. This simple circumstance makes the calculation of negative rules computationally much more challenging compared to positive rules and necessitates the application of much stricter thresholds on the rules of interest. While analyzing rule strength distribution we considered only the rules exhibiting strength higher than 0.1. The number of weaker rules (strength below 0.1) is too high due to the combinatorial explosion caused by random feature combinations, making their analysis computationally prohibitive. However, even in the strength interval 0.1 – 1.0 the number of negative rules is several orders of magnitude higher that the number of positive rules. To make the task computationally tractable we additionally imposed a threshold on minimal leverage which effectively helps to select only the most 'non-random' rules (see Methods) and eliminates all rules with the strength below 0.97. The distributions of negative rule strength with different minimal coverage counts are plotted in Figure 1.

The fraction of proteins in the PEDANT database constituting exceptions from strong rules in the strength interval between 0.97 and 1.0 as well as the fraction of relevant (homology-transferred) features participating in such rules is very low. In total, we identified 6875 features (0.8%) in the annotation of 1031 proteins (1.9%) as potential annotation errors.

### Analysis of potentially erroneous annotation features

#### Taxon-specific rules

**Figure 2:**
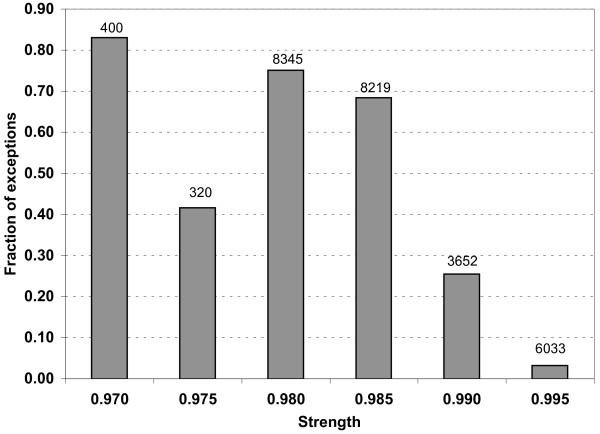
**Fraction of annotation terms corrected based on the taxonomic information among all rule exceptions**. The number of all exceptions found in each strength interval is shown above each bar.

In order to estimate the number of actual annotation errors among exceptions from strong rules the first test was designed to exclude the influence of taxon specificity. It turned out that a very large number of rules combined FunCat labels on one side of the rule with the taxon of the protein origin on the other side (we used here only the highest-level taxons, namely Eukaryota, Bacteria, Archae, and Viruses). There were 3159 rules (33% of all negative rules) with such structure. Because many FunCat labels are taxon-specific (see Methods), these labels should ideally only be present in the annotation of the genes belonging to the corresponding taxa; homology-based transfer of such annotation attributes is highly prone to error. Where a taxonomically specific FunCat label is incompatible with the known gene taxon, it is the FunCat assignment which is guaranteed to be erroneous, since the protein origin is doubtlessly known. This simple test resulted in automatic correction of almost 50% of all exceptions in our set of strong negative rules. Figure 2 shows how the fraction of corrected exceptions among all exceptions depends on the rule strength.

#### Performance of the method

After the filter for taxonomy-specific FunCat labels was implemented, the set of 6432 rules formed all negative rules for our annotation sample. These rules involved 4687 transferable features (0.6% of all features) in the annotation of 822 proteins (1.5% of all proteins).

To estimate the prevalence of errors among exceptions not corrected by the taxonomy procedure described above we selected randomly a sample of 100 rules and analyzed their exceptions manually. In 96% of examined exceptions at least one of the features constituting the rule was assigned wrongly to the given protein. The overall specificity of the approach was estimated to be as high as 98%: practically all feature combinations associated with exceptions included at least one annotation error.

**Figure 3:**
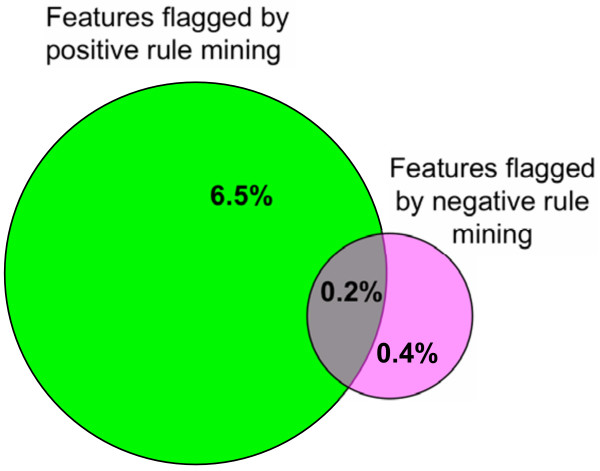
**Coverage of the negative and positive rule mining approaches**. The numbers represent the percentage of all annotation features identified as potentially erroneous by each individual method and by both of them

The specificity of the negative rules is thus much higher than that in the case of positive rules [18] which was estimated to be around 68% based on careful manual verification. By design, exceptions from negative association rules can only reveal over-annotation, i.e. erroneous assignment of attributes to protein entries, while under-annotation (missing attributes) cannot be detected. While manually curated databases are typically under-annotated and this approach is not efficient for them (the performance of the method was very low when tested on the Swiss-Prot database, data not shown), over-annotation is a typical problem of many automatic software pipelines, including PEDANT, and the ability to correct this type of errors using negative rule mining is valuable. At the same time, the approach based on exceptions from strong negative rules yields much smaller coverage than positive rule mining. As seen in Figure 3, negative rule mining allows identifying 11 times fewer annotation features (0.6% for negative rules *versus *6.7% for positive rules) that participate in incompatible feature combinations. More than two thirds of these features do not get detected by positive rule mining.

#### What fraction of the annotation features flagged by the negative rules are actual annotation errors?

Our approach is designed to flag incompatible feature combinations for subsequent manual inspection rather than to automatically correct annotation errors in an unsupervised fashion. With the exception of taxon-specific rules where FunCat labels incompatible with the taxonomic origin of a protein are guaranteed to be errors, we do not know exactly which feature of a flagged feature combination is wrong. Besides there always exists a chance that all features constituting an exception from a strong negative rule are nevertheless correctly assigned and that the exception is in fact biologically motivated.

It would be desirable to validate our predictions against high-quality manually curated databases such as Swiss-Prot or BRENDA [29], but this, unfortunately cannot be done at sufficiently large scale. As discussed above most of the annotation errors found in PEDANT are over-predictions which can not easily be confirmed by comparison with Swiss-Prot entries as the latter tend to be under-annotated. If a certain feature is present in a Swiss-Prot entry, it is almost certainly correct, however, if a feature is absent no statement can generally be made. BRENDA focuses on one aspect of protein annotation – EC numbers – providing detailed classification of enzymes at four hierarchical levels. Correspondingly, there are only few proteins associated with each four digit EC number while association rule mining relies on frequent annotation items. In this study rules were required to have coverage count of at least 100, and only six EC numbers satisfied this condition.

We therefore attempted to estimate the fraction of actual annotation errors among those features flagged as suspicious and, for comparison, in non-flagged features by careful manual inspection as described in *Methods*. As seen in Table 2, features flagged as suspicious are almost 1.4 fold enriched in actual annotation errors than the unmarked ones.

**Table 2:** Manual verification of randomly selected feature samples

	Number of verified terms	Classified as "errors"	Percentage of actual errors in the sample
Flagged features	203	150	74%
Non-flagged features	798	430	54%

## Conclusion

Based on our assessment it becomes apparent that almost all incompatible feature combinations found by negative association rule mining include at least one wrongly assigned annotation term. The fraction of individual features flagged as suspicious is about 0.6% from the total number of features assigned by PEDANT and it is significantly enriched in annotation errors. Moreover, roughly two thirds of such erroneous assignments are not identified by positive rule mining. We conclude that applying a combination of positive rule mining described earlier [18] and negative rule mining presented creates an opportunity to enhance the fidelity of genome annotation in two alternative ways. First, insights about the sources of annotation errors gained in this investigation can be used to adjust the automatic annotation pipeline in order to minimize generation of these errors in the future. Examples of such possible modifications include taxon-specific homology-based transfer of functional categories and utilization of individualized similarity thresholds for various features. Second, suspicious features can be visually marked for subsequent inspection by the user. While this approach is better suited for manually curated databases where errors actually get corrected by human experts, it is also useful for automatic systems such as PEDANT where users get alerted to specific less trusted annotation items that should be used with caution.

## Authors' contributions

IIA and DF conceived the study. IIA designed and executed the analysis of negative rules and rule statistics. GF conducted manual verification of predicted annotation errors. DF supervised the project. All authors participated in the drafting and revising of the manuscript, and read and approved the final manuscript.

## Supplementary Material

Additional file 1 **The set of all negative rules used in this work**. Rules.out Click here for file

## References

[B1] Consortium TUP (2007). The Universal Protein Resource (UniProt). Nucleic Acids Res.

[B2] Brown D, Sjolander K (2006). Functional classification using phylogenomic inference. PLoS Comput Biol.

[B3] Metzker ML (2005). Emerging technologies in DNA sequencing. Genome Res.

[B4] Bork P, Bairoch A (1996). Go hunting in sequence databases but watch out for the traps. Trends Genet.

[B5] Galperin MY, Koonin EV (1998). Sources of systematic error in functional annotation of genomes: domain rearrangement, non-orthologous gene displacement and operon disruption. In Silico Biol.

[B6] Guigo R, Reese MG (2005). EGASP: collaboration through competition to find human genes. Nat Methods.

[B7] Durbin R, Eddy SR, Krogh A, Mitchison G (1999). Biological Sequence Analysis: Probabilistic Models of Proteins and Nucleic Acids.

[B8] Jones DT (1999). Protein secondary structure prediction based on position-specific scoring matrices. J Mol Biol.

[B9] Gardy JL, Brinkman FS (2006). Methods for predicting bacterial protein subcellular localization. Nat Rev Microbiol.

[B10] Levy ED, Ouzounis CA, Gilks WR, Audit B (2005). Probabilistic annotation of protein sequences based on functional classifications. BMC Bioinformatics.

[B11] Kanehisa M, Goto S, Hattori M, Aoki-Kinoshita KF, Itoh M, Kawashima S, Katayama T, Araki M, Hirakawa M (2006). From genomics to chemical genomics: new developments in KEGG. Nucleic Acids Res.

[B12] Guldener U, Munsterkotter M, Oesterheld M, Pagel P, Ruepp A, Mewes HW, Stumpflen V (2006). MPact: the MIPS protein interaction resource on yeast. Nucleic Acids Res.

[B13] Andreeva A, Howorth D, Brenner SE, Hubbard TJ, Chothia C, Murzin AG (2004). SCOP database in 2004: refinements integrate structure and sequence family data. Nucleic Acids Res.

[B14] Consortium GO (2006). The Gene Ontology (GO) project in 2006. Nucleic Acids Res.

[B15] Ruepp A, Zollner A, Maier D, Albermann K, Hani J, Mokrejs M, Tetko I, Guldener U, Mannhaupt G, Munsterkotter M, Mewes HW (2004). The FunCat, a functional annotation scheme for systematic classification of proteins from whole genomes. Nucleic Acids Res.

[B16] Finn RD, Mistry J, Schuster-Bockler B, Griffiths-Jones S, Hollich V, Lassmann T, Moxon S, Marshall M, Khanna A, Durbin R, Eddy SR, Sonnhammer EL, Bateman A (2006). Pfam: clans, web tools and services. Nucleic Acids Res.

[B17] Letunic I, Copley RR, Pils B, Pinkert S, Schultz J, Bork P (2006). SMART 5: domains in the context of genomes and networks. Nucleic Acids Res.

[B18] Artamonova II, Frishman G, Gelfand MS, Frishman D (2005). Mining sequence annotation databanks for association patterns. Bioinformatics.

[B19] Zhang C, Zhang S (2002). Association rule mining: models and algorithms.

[B20] Frishman D, Albermann K, Hani J, Heumann K, Metanomski A, Zollner A, Mewes HW (2001). Functional and structural genomics using PEDANT. Bioinformatics.

[B21] Blumenthal T, Evans D, Link CD, Guffanti A, Lawson D, Thierry-Mieg J, Thierry-Mieg D, Chiu WL, Duke K, Kiraly M, Kim SK (2002). A global analysis of Caenorhabditis elegans operons. Nature.

[B22] Riley ML, Schmidt T, Artamonova II, Wagner C, Volz A, Heumann K, Mewes HW, Frishman D (2007). PEDANT genome database: 10 years online. Nucleic Acids Res.

[B23] Mulder NJ, Apweiler R, Attwood TK, Bairoch A, Bateman A, Binns D, Bork P, Buillard V, Cerutti L, Copley R, Courcelle E, Das U, Daugherty L, Dibley M, Finn R, Fleischmann W, Gough J, Haft D, Hulo N, Hunter S, Kahn D, Kanapin A, Kejariwal A, Labarga A, Langendijk-Genevaux PS, Lonsdale D, Lopez R, Letunic I, Madera M, Maslen J, McAnulla C, McDowall J, Mistry J, Mitchell A, Nikolskaya AN, Orchard S, Orengo C, Petryszak R, Selengut JD, Sigrist CJ, Thomas PD, Valentin F, Wilson D, Wu CH, Yeats C (2007). New developments in the InterPro database. Nucleic Acids Res.

[B24] Bork P (2000). Powers and pitfalls in sequence analysis: the 70% hurdle. Genome Res.

[B25] Agrawal R, Srikant R (1994). Fast Algorithms for Mining Association Rules. Proc 20th Int Conf Very Large Data Bases, VLDB.

[B26] Wu X, Zhang C, Zhang S (2004). Efficient mining of both positive and negative association rules. ACM Transactions on Information Systems (TOIS).

[B27] Borgelt C, Kruse R (2002). Induction of Association Rules: Apriori Implementation. 15th Conference on Computational Statistics.

[B28] Mewes HW, Albermann K, Heumann K, Liebl S, Pfeiffer F (1997). MIPS: a database for protein sequences, homology data and yeast genome information. Nucleic Acids Res.

[B29] Schomburg I, Chang A, Ebeling C, Gremse M, Heldt C, Huhn G, Schomburg D (2004). BRENDA, the enzyme database: updates and major new developments. Nucleic Acids Res.

